# Bioactive Flavonoids from *Paulownia tomentosa* Flowers: Extraction Optimization and α-Glucosidase Inhibitory Kinetics

**DOI:** 10.3390/foods14223941

**Published:** 2025-11-18

**Authors:** Fu Jiang, Haibo Yang, Xiaoqiao Zhai, Zhenli Zhao, Guoqiang Fan

**Affiliations:** 1 Institute of Paulownia, Henan Agricultural University, Zhengzhou 450002, China; 18673700016@163.com (F.J.); haiboyang2277@163.com (H.Y.); zhaozhl2006@126.com (Z.Z.); 2 Henan Province Academy of Forestry, Zhengzhou 450008, China

**Keywords:** *Paulownia tomentosa*, flavonoids, α-glucosidase, macroporous resin, response surface methodology

## Abstract

*Paulownia tomentosa* flowers are rich in flavonoids with promising biological activities. However, few studies have investigated their potential for α-glucosidase inhibition. This study compared ultrasound-assisted cellulase extraction and ultrasound-assisted aqueous two-phase extraction for the recovery of flavonoids from *Paulownia tomentosa* flowers. The aqueous two-phase extraction method demonstrated superior performance, with optimal conditions determined as 17.80% (NH_4_)_2_SO_4_, 12 min ultrasonication, and 44 °C. Purification was efficiently achieved using NKA-9 macroporous resin. Scanning electron microscopy revealed that ultrasonic treatment disrupted the cell walls, facilitating flavonoid release. Ultra-performance liquid chromatography–tandem mass spectrometry identified apigenin-7-glucuronide and scutellarin as the predominant flavonoids. Notably, several compounds—including scutellarin, ombuin, robinetin, and astragalin—were reported for the first time in this plant. The extracted flavonoids exhibited significant α-glucosidase inhibitory activity, with an IC_50_ value of 0.412 mg/mL, and showed mixed-competitive inhibition. Luteolin 7-O-glucuronide was identified as a major active constituent, exhibiting stronger inhibition than the total flavonoids while sharing the same mechanism. These findings establish a theoretical foundation for the efficient and sustainable extraction of *P. tomentosa* flavonoids and support their further development for pharmaceutical applications.

## 1. Introduction

*Paulownia* is a perennial woody plant in the genus *Paulownia* and family *Paulowniaceae* [1]. The flowers of *Paulownia* are often used in Chinese herbal medicine to treat inflammation and bacterial infections, and they have a good taste, unique flavor, and medicinal properties. Furthermore, *Paulownia tomentosa* flowers are recognized for their edibility, pleasantly sweet taste, unique aroma, and documented use in traditional medicine [2]. Additionally, *Paulownia* flowers are promising for natural enhancement of animal immune systems [3]. *Paulownia* is mainly cultivated for its wood to make furniture and musical instruments, and few applications using the biological activities of *Paulownia* flowers have also been observed. Recently, further research on *Paulownia* flowers has led to progress in the study of their biological activity. The flavonoid content in *Paulownia* flowers is high, and these flavonoids have good antioxidant and antibacterial activities in vitro [4]. Flavonoids are known to exhibit strong antioxidant activity through free radical scavenging, as well as anti-inflammatory, antimicrobial, and anti-cancer properties. Additionally, flavonoids can ameliorate health issues caused by a high-fat diet, including hyperlipidemia, fatty liver disease, and insulin resistance, through the AMP-activated protein kinase pathway [5].

Ultrasonic-assisted extraction (UAE) was utilized to leverage its cell wall-disrupting capability, which is driven by acoustic cavitation and its resultant mechanical and thermal actions [6], for the efficient liberation of flavonoids from *Paulownia tomentosa* flower tissues. Cellulase extraction is characterized by mild conditions, straightforward operation, and high efficiency [7]. Aqueous two-phase extraction (ATPE) is suitable for the extraction of low-molecular-weight compounds and can improve the extraction yield and maintain the activities of the compounds [8]. Current extraction methods for *Paulownia* flower flavonoid (PFF) have been predominantly focused on microwave- or ultrasound-assisted techniques, while the application of ultrasound-assisted cellulase extraction (UA-CE) combined with ultrasound-assisted aqueous two-phase extraction (UA-ATPE) has been less extensively investigated for PFF isolation. Microporous resins, characterized by their environmental compatibility, low cost, high adsorption efficiency, and mild processing requirements [9], have proven effective for flavonoid purification.

α-Glucosidase, which is involved in carbohydrate metabolism, catalyzes the hydrolysis of α-1,4-glycosidic bonds to release α-glucose from the non-reducing ends of oligosaccharides [10]. Effective α-glucosidase inhibitors can delay carbohydrate digestion and reduce postprandial hyperglycemia [11]. Diabetes mellitus, a chronic metabolic disorder, requires long-term pharmacological intervention for glycemic control [12]. Clinically used inhibitors such as acarbose, miglitol, and sitagliptin, while effective in suppressing α-glucosidase activity, are associated with adverse gastrointestinal effects including flatulence and diarrhea [13]. Therefore, finding new natural plant-derived α-glucosidase inhibitors with minor side effects is of significant importance. To date, the inhibitory effects of *Paulownia* flavonoids on α-glucosidase activity have not been reported, making the investigation of α-glucosidase inhibition by flavonoids from *P. tomentosa* flowers particularly valuable for research.

Despite the known abundance of flavonoids in *Paulownia tomentosa* flowers, a significant knowledge gap remains regarding the development of efficient extraction techniques and the systematic evaluation of their α-glucosidase inhibitory potential. In this study, a combination of single-factor experiments and response surface methodology was employed to optimize the PFF extraction process. The inhibitory kinetics against α-glucosidase were investigated for both PFF and its principal flavonoid constituents. The primary objective of this study was to develop an efficient and sustainable method for extracting bioactive flavonoids with high α-glucosidase inhibitory activity from PFF and provide a scientific basis for its industrial application and medicinal development.

## 2. M aterials and Methods

### 2.1. Chemicals and Materials

#### 2.1.1. Sample Pretreatment

*P. tomentosa* flowers were collected from a Paulownia cultivation base in Zhongmu County (Zhengzhou, China). The flowers were dried under natural conditions in a shaded, well-ventilated environment at ambient temperature (approximately 25 °C) until a constant weight was achieved, typically for 5–7 days, to standardize the drying endpoint. The dried material was then ground and sieved through a 60-mesh sieve (particle size ≤ 0.3 mm) for subsequent use.

#### 2.1.2. Materials and Reagents

Cellulase (enzyme activity: 50 U/mg), rutin (purity > 95%), ammonium sulfate, α-glucosidase (enzyme activity: 250 U, 50 U/mg), *p*-nitrophenyl-α-d-glucopyranoside (purity ≥ 99%), and acarbose (purity ≥ 98%) were purchased from Shanghai Yuanye Biotechnology Co. (Shanghai, China). NKA-9, HPD-100, HPD-300, NKA-2, HPD-722, D101, and AB-8 macroporous resins were purchased from Solarbio (Beijing, China). Anhydrous ethanol, sodium nitrite, sodium hydroxide, aluminum nitrate, anhydrous sodium carbonate and hydrochloric acid are all of analytical purity and reagent grade, and they are sourced from Sinopharm Group Chemical Reagents Company (Shanghai, China).

### 2.2. Optimization of UA-Cellulase Extraction for PFF

#### 2.2.1. Rutin Standard Curve Production

Rutin standard solutions at varying concentrations were prepared, with distilled water serving as the blank control. The sodium nitrite–aluminum nitrate–sodium hydroxide chromogenic method [14] was employed to measure absorbance at 510 nm, and the obtained values were utilized to construct the rutin standard curve.

#### 2.2.2. Determination of Flavonoid Content in Extracts

The absorbance was determined by the sodium nitrite–aluminum nitrate–sodium hydroxide chromogenic method [14], and the mass concentration of total flavonoids was obtained from the rutin standard curve. The extraction rate of total flavonoids from *P. tomentosa* flowers (*Y*) was calculated using the following equation:

(1) *Y* = *C* × 25 × 10/*M*

where *M* is the mass of the *P. tomentosa* flower powder, and *C* is the mass concentration of flavonoids in the extract from the *P. tomentosa* flowers.

#### 2.2.3. One-Factor and Response Surface Optimization Experiments

The extraction process was optimized through a structured experimental design involving single-factor experiments. The initial extraction was performed with cellulase as an additive at a solid–liquid ratio of 1:15 (*w*/*v*) under ultrasonication (40 °C, 280 W). Key factors, including ethanol concentration, cellulase concentration, digestion time, digestion temperature, and ultrasonication time, were evaluated sequentially across predetermined levels to determine their impact on flavonoid yield (Table S1). The Box–Behnken design (BBD) for RSM was used to optimize the extraction conditions of total flavonoids from *P. tomentosa* flowers. The effects of the ethanol concentration (*A*), cellulase concentration (*B*), and ultrasonic time (*C*) on the extraction of total flavonoids from *P. tomentosa* flowers were studied (Table S2).

### 2.3. Optimization of UA-ATPE for PFF

#### 2.3.1. Preparation of Double Aqueous Phase Diagram

The ethanol–ammonium sulfate aqueous two-phase system (ATPS) was selected based on its well-established efficiency in the separation of bioactive compounds, particularly flavonoids. Ammonium sulfate is widely used as a phase-forming salt due to its high solubility in water, strong salting-out effect, and low cost. The phase diagram of an ethanol–ammonium sulfate aqueous system was determined by the turbidity titration method [15]. This method involves the dropwise addition of a titrant (e.g., water) to a turbid, biphasic mixture until the point of phase clarification, allowing for the accurate determination of the binodal curve. Ammonium sulfate was dissolved in distilled water. Ethanol was added under ultrasonication until turbidity appeared, followed by dropwise water addition until clarification, with volumes recorded. Repeated cycles determined phase transition solvent ratios. Ethanol/water mass fractions were calculated to plot the phase diagram.

#### 2.3.2. Determination of Flavonoid Content in Extracts

The absorbance was determined by the sodium nitrite–aluminum nitrate–sodium hydroxide chromogenic method [14], and the mass concentration of total flavonoids was obtained from the rutin standard curve. The extraction rate of total flavonoids from *P. tomentosa* flowers (*Y*) was calculated using the following equation:

(2) *Y* = *C* × *V* × *n*/*M*

where *C* is the flavonoid concentration in the upper phase in the extraction (mg/mL), *V* is the volume of the upper phase in the extraction (mL), *n* is a dilution factor, and *M* is the mass of the *P. tomentosa* flower powder (mg).

#### 2.3.3. One-Factor and Response Surface Optimization Experiments

The ultrasound-assisted aqueous two-phase extraction (UA-ATPE) process was systematically optimized. An ATPS was constructed with ethanol and ammonium sulfate, employing a solid–liquid ratio of 1:30 (*w*/*v*). The optimization followed a sequential single-factor experimental design, where the effects of four key parameters on flavonoid yield were investigated. The specific factors and their tested levels are summarized in Table S3. The BBD RSM was used to optimize the extraction conditions of total flavonoids from *P. tomentosa* flowers. The effects of the concentration of (NH_4_)_2_SO_4_ (*A*), ultrasonic time (*B*), and ultrasonic temperature (*C*) on the extraction of total flavonoids from *P. tomentosa* flowers were studied (Table S4).

### 2.4. Optimization of PFF Purification

#### 2.4.1. Pretreatment of Macroporous Resins

All macroporous resins were first soaked in 95% ethanol for 12 h with constant agitation, and then washed with deionized water until odorless. Subsequently, the macroporous resins were soaked in 4% hydrochloric acid for 4 h, with continuous stirring to keep the resin in contact with the hydrochloric acid solution. The resins were then washed with deionized water until neutral. Finally, the resins were soaked in 4% NaOH for 4 h, rinsed with deionized water until neutral, and stored at −4 °C.

#### 2.4.2. Screening of Macroporous Resins

Adsorption/desorption tests were conducted on 7 macroporous resins. The macroporous resins were accurately weighed and placed in separate 100 mL Erlenmeyer flasks, to which 20 mL of total flavonoids extract of *P. tomentosa* flower was added. The flasks were heated at 25 °C with rotation at 140 rpm. Static adsorption was conducted for 24 h. After adsorption equilibrium was reached, the flavonoids attached to the resin surface were rinsed with ultrapure water, 20 mL of 70% ethanol was added, and desorption was performed at 140 rpm for 24 h. The adsorption rates (*A*, %) and desorption rates (*D*, %) of different resins were calculated as follows:

(3) Adsorption capacity: *Q*_e_ = (*C*_0_ − *C*_1_) × *V*/*M*

(4) Adsorption rate: *A* = (*C*_0_ − *C*_1_)/*C*_0_ × 100

(5) Desorption capacity: *Q*_d_ = *C*_2_ × *V*_2_/*M*

(6) Desorption rate: *D* = *C*_2_/(*C*_0_ − *C*_1_) × 100

where *C*_0_, *C*e, and *C*_2_ (mg/mL) are the concentration of total flavonoids in the pre-adsorption, at adsorption equilibrium, and in the desorption solution, respectively; *V* and *V*_2_ (mL) are the volume of the solution before and after adsorption, respectively; *Q*_e_ and *Q*_d_ (mg/g) are adsorption equilibrium and desorption equilibrium, respectively; and *M* (g) is the wet mass of the macroporous resin.

#### 2.4.3. Determination of Leakage Curve, Washing Volume, and Elution Profile

A 2 mg/mL PFF solution was subjected to dynamic adsorption at 1 mL/min, with effluent collected at 10 min intervals. Flavonoid concentrations were measured to plot the leakage curve. Post-adsorption, the resin was washed with distilled water until effluent became colorless, establishing a water consumption curve for optimal washing volume determination. Subsequent elution with 70% ethanol (1.5 mL/min) yielded fractions analyzed at 10 min intervals to construct the elution curve.

#### 2.4.4. One-Factor Experiments

The initial extract of PFF was purified using macroporous resin. The sample volume was 60 mL, the volume of water was 130 mL, and the eluent volume was 150 mL. The effects of the PFF concentration (2, 3, 4, 5, 6 mg/mL) on the purification effect of the macroporous resin was investigated. PFF flow rate (1, 1.5, 2, 2.5, 3 mL/min), ethanol concentration (40%, 50%, 60%, 70%, 80%), and ethanol flow rate (1, 1.5, 2, 2.5, 3 mL/min) were studied sequentially.

#### 2.4.5. Purification Effect of Macroporous Resin

After the crude extract of total flavonoids of *P. tomentosa* flower was purified by the NKA-9 macroporous adsorption resin, the eluent was reduced on a rotary evaporator until it had no alcohol odor. A total flavonoid powder was then obtained by freeze-drying. The powder (10 mg) was accurately weighed, dissolved in 70% ethanol, and the volume was reduced to 10 mL. The total flavonoid content of purified *P. tomentosa* flowers was determined. The method for analyzing unpurified crude extracts was the same as above.

### 2.5. UV–Vis and FT-IR Spectroscopy

Purified total flavonoid powder from *P. tomentosa* flowers (10 mg) was accurately weighed and placed in a 10 mL volumetric flask with 70% ethanol. After diluting all the samples five-fold, they were analyzed by UV–Vis spectrophotometry at 200–500 nm. Ethanol (70%) was used as the reference solution.

For FTIR spectroscopy, 1.0 mg of *P. tomentosa* flowers total flavonoid purified powder and 100 mg of KBr powder were accurately weighed into a mortar and ground until homogenized. The powder was pressed into uniform and transparent flakes. Blank KBr was used as the background. The sample was analyzed by FTIR spectroscopy from 400 to 4000 cm^−1^. The resolution was 4 cm^−1^ and the number of scans was 16.

### 2.6. Scanning Electron Microscopy (SEM) Analysis

*P. tomentosa* flower powders extracted via UA-CE and ethanol–ammonium sulfate ATPS were centrifuged (8000 rpm, 10 min) to collect pellets. After filtration and ultrapure water washing, both extracted and untreated powders were oven-dried at 50 °C to constant mass. Gold-sputtered samples were analyzed by SEM.

### 2.7. UPLC-MS/MS Analysis

Column: HSS T3 (100 × 2.1 mm, 1.8 μm; Waters). The mobile phase was a mixture of water containing 0.1% formic acid (phase A) and methanol containing 0.1% formic acid (phase B) at a flow rate of 0.3 mL/min. Column temperature: 40 °C. Injection volume: 2 μL. A Q Exactive HFX high-resolution mass spectrometer (Thermo Fisher Scientific, Waltham, MA, USA) was used to collect primary and secondary spectra. The mass spectrometer used electrospray ionization. Sheath gas, 40 arb; auxiliary gas, 10 arb; ion spray voltage, −2800 V; temperature, 350 °C; and ion transfer tube temperature, 320 °C. A full scan was performed in data-dependent MS2 mode using positive ions for the range *m/z* 70–1050. We used standard substances such as kaempferol, apigenin, luteolin and rutin for component identification.

### 2.8. Inhibition of α-Glucosidase by PFF and Flavonoid Monomer Substances

An α-glucosidase inhibition assay was performed using a modification of an established method [10]. 0.1 M PBS was initially added to the 96-well plate, followed by the sequential addition of total flavonoid solutions (Apigenin-7-glucuronide, Kaempferol 3-O-sophoroside and Luteolin 7-O-glucuronide) at varying concentrations and 0.1 U/mL α-glucosidase solution. After 30 min of incubation (37 °C), 20 μL pNPG (1.2 mg/mL) was added, followed by 40 min incubation and reaction termination with 80 μL Na_2_CO_3_ (0.2 mol/L). Absorbance was measured at 405 nm, using PBS as blank and acarbose as positive control. The α-glucosidase inhibition rate was calculated, with IC_50_ values determined using GraphPad Prism.

(7) $$\alpha \text{-}\mathrm{glucosidase}\;\mathrm{inhibition}\;\mathrm{rate}\;(\%)=\left(1-\frac{A1-A2}{A3-A4}\right)\times 100\%$$

### 2.9. Reversibility of Inhibition of α-Glucosidase by PFF and Luteolin 7-O-Glucuronide

Reversibility of inhibition was studied using a slight modification of an established method [16]. The 96-well plate was loaded with PBS, total flavonoid solutions (Luteolin 7-*O*-glucuronide) at varying concentrations, and α-glucosidase solution. After 20 min of incubation at 37 °C, 20 μL of 1.2 mg/mL pNPG solution was added. The absorbance at 405 nm was measured immediately and then at 1 min intervals. The initial reaction rate of α-glucosidase was plotted against the concentration of α-glucosidase.

### 2.10. Type of Inhibition of α-Glucosidase by PFF and Luteolin 7-O-Glucuronide

0.1 M PBS and total flavonoid solutions (Luteolin 7-O-glucuronide) at varying concentrations were added to the 96-well plate, followed by the addition of α-glucosidase solution (0.1 U/mL). After 20 min of incubation at 37 °C, 20 μL of pNPG solution (0.4, 0.8, 1.2, 1.6, and 2.0 mg/mL) was added to each well. The absorbance at 405 nm was measured immediately and then at intervals of 1 min. The type of inhibition was determined using kinetic curves and Lineweaver–Burk plots [17].

### 2.11. Statistical Analysis

All experimental data were expressed as mean ± SEM (standard error of mean). Statistical significance (*p* < 0.05) was determined by one-way ANOVA followed by Duncan’s multiple comparison test using SPSS 25.0. The response surface optimization and ANOVA variance analysis were conducted using Design-Expert 8.0.6 11.0 (Stat-Ease Inc., Minneapolis, MN, USA), and the interaction effects were evaluated, while kinetic parameters and IC_50_ values were derived from nonlinear regression analysis using GraphPad Prism 8.0.

## 3. Results and Discussion

### 3.1. Optimization of UA-Cellulase Extraction for PFF

#### 3.1.1. Single-Factor Experiments

**Figure 1:**
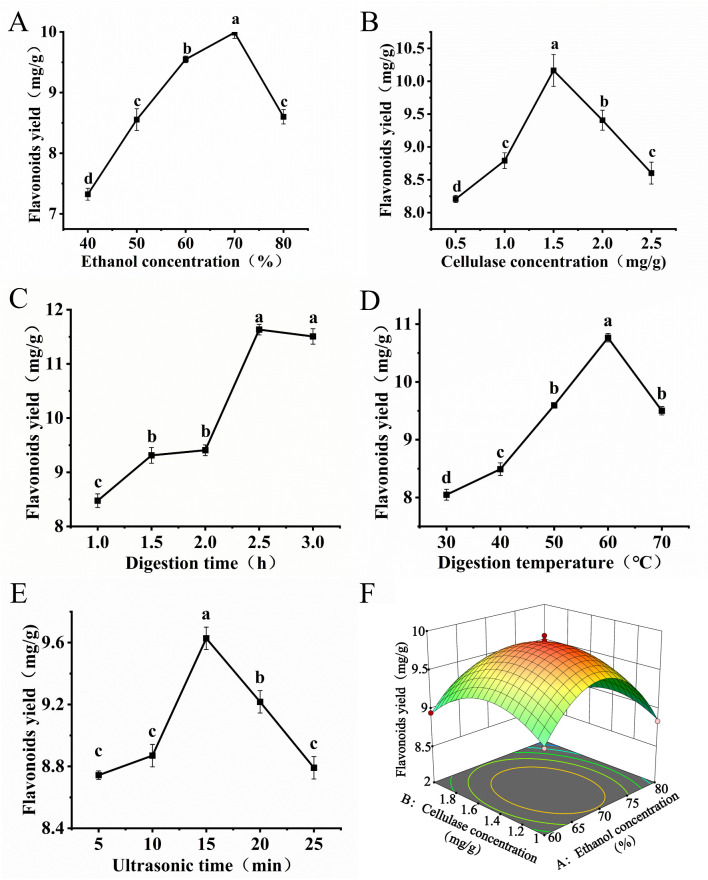
Effects of a single factor and RSM on the flavonoid yield from *P. tomentosa* flowers. (**A**) Effects of ethanol concentration on PFF extraction yield. (**B**) Effects of cellulase concentration on PFF extraction yield. (**C**) Effects of digestion time on PFF extraction yield. (**D**) Effects of digestion temperature on PFF extraction yield. (**E**) Effects of ultrasonic time on PFF extraction yield. (**F**) The 3D response surface plot of UA-CE. Different lowercase letters indicate statistically significant differences (*p* < 0.05), while the absence of letters denotes no statistically significant difference (*p* > 0.05).

An excessive ethanol concentration may reduce flavonoid solubility because of altered solvent polarity and thereby decrease the extraction efficiency (Figure 1A). A high cellulase concentration will enhance cell wall disruption, which will promote release of intracellular non-flavonoid substances (Figure 1B). This phenomenon likely compromises the extraction specificity and contributes to the subsequent yield reduction [18]. Moderate increases in the digestion time and temperature enhance cellulase activity (Figure 1C,D). However, a prolonged digestion time or excessively high temperature may induce enzyme denaturation or promote co-extraction of non-target compounds, which will reduce the extraction specificity [19]. The observed biphasic yield profile is attributed to a shift in the dominant ultrasonic mechanism. Initially, the extraction yield is enhanced by cavitation-induced cell wall disintegration, which accelerates mass transfer. Beyond the optimum point, the cumulative thermal effect of prolonged ultrasonication becomes predominant, potentially degrading flavonoids and counteracting the initial gains from improved cell disruption [20].

#### 3.1.2. Response Surface Optimization Experiments

The results are summarized in Table S5. A quadratic polynomial regression model was established through multivariate analysis and expressed as follows:

(8) *Y* = 9.87 − 0.1618*A* − 0.1263*B* + 0.0700*C* − 0.0513*AB* + 0.0355*AC* − 0.0197*BC* − 0.6601*A*^2^ − 0.3681*B*^2^ − 0.1630*C*^2^

where *Y* is the total flavonoid yield (mg/g), *A* is the ethanol concentration (%), *B* is the cellulase concentration (mg/g), and *C* is the ultrasonic time (min).

The quadratic regression model exhibited exceptional statistical validity with an F-value of 204.51 and a highly significant *p*-value (<0.01) (Table S6). Furthermore, the model demonstrated outstanding goodness-of-fit, as evidenced by the coefficient of determination (*R*^2^ = 0.996) and adjusted *R*^2^ (*R*^2^_Adj_ = 0.991). The adjusted coefficient of determination is 0.991, which indicates that the selected factors have a significant impact on the PFF output.

Among the model terms, the linear coefficients (*A*, *B*, and *C*), quadratic terms (*A*^2^, *B*^2^, and *C*^2^), and the *AB* interaction exhibited significant effects (*p* < 0.01) for all terms except *AB* (*p* < 0.05). The elliptical and tightly packed contours of the *AB* interaction (Figure 1F and Figure S2) indicated that there was a pronounced synergistic effect between these two factors [21]. The steep curvature of the response surface further corroborated the statistically significant interaction between *A* and *B*, which aligned with the ANOVA results (*p* < 0.05 for *AB*).

#### 3.1.3. Model Validation

The optimum extraction parameters determined through RSM were an ethanol concentration of 69%, cellulase concentration of 1.4 mg/g, and ultrasonication time of 16 min for model validation. An average flavonoid content of 9.896 mg/g was obtained from three replicate experiments performed under these conditions, which was close to the theoretical value (relative error < 0.03%). Yin et al. used RSM to optimize the conditions for extraction of flavonoids from *Equisetum* using cellulase [19]. The amount of cellulase they used was high, and the flavonoid yield was only 4.88 mg/g. By comparison, in this study, we used less cellulase and ethanol and the extraction yield was higher (9.896 mg/g) because of the ultrasonication. Therefore, our results show that the flavonoid yield can be effectively increased by UAE.

### 3.2. Optimization of UA-ATPE for PFF

#### 3.2.1. Phase Diagram and Single-Factor Experiments

**Figure 2:**
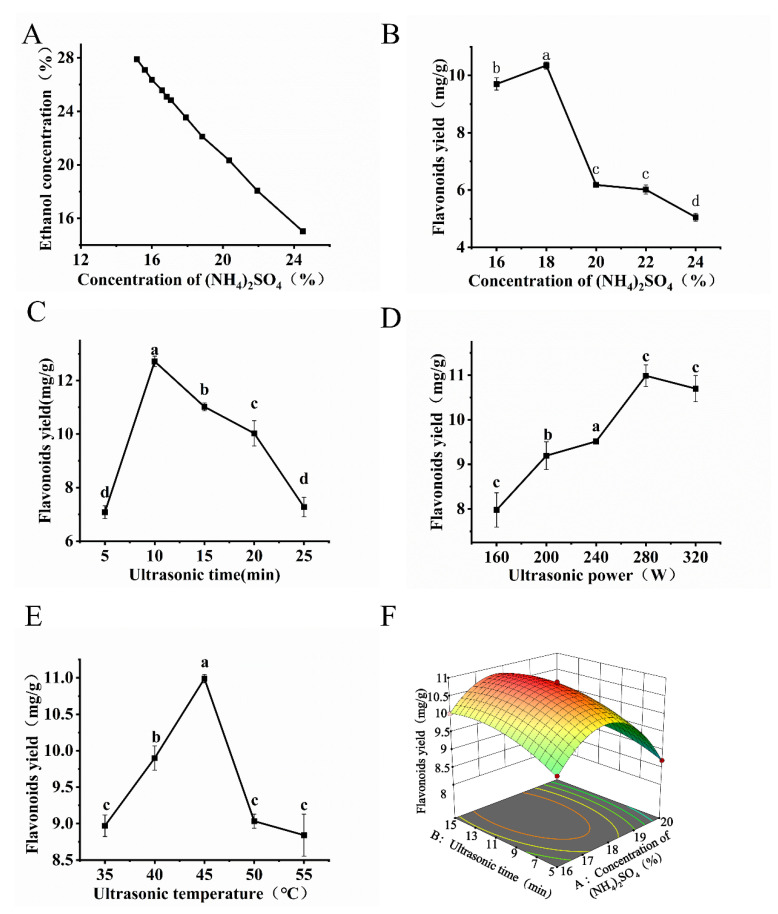
Effects of a single factor and RSM on the flavonoid yield from *P. tomentosa* flowers. (**A**) Aqueous two-phase phase diagram. (**B**) Effects of concentration of (NH_4_)_2_SO_4_ on PFF extraction yield. (**C**) Effects of ultrasonic time on PFF extraction yield. (**D**) Effects of ultrasonic power on PFF extraction yield. (**E**) Effects of ultrasonic temperature on PFF extraction yield. (**F**) The 3D response surface plot of UA-ATPE. The presence of different letters indicates that the observed differences are statistically significant (*p* < 0.05).

The phase diagram of the ATPS was plotted (Figure 2A). As the concentration of (NH_4_)_2_SO_4_ increased, the yield of total flavonoids initially increased and then decreased (Figure 2B). The mass fraction of ammonium sulfate determines the partition coefficient of organic solvent and salt between the two phases, and this affects the flavonoid extraction efficiency [22]. The extraction yield exhibited a biphasic dependence on ultrasonication time, power, and temperature (Figure 2C–E). The initial increase corresponds to a kinetics-controlled regime where cavitation-induced cell wall disruption enhances mass transfer of flavonoids. Beyond optimal conditions, yield reduction occurs due to mechanistic shift: prolonged exposure promotes thermal degradation of flavonoids [21], while excessive power causes structural fragmentation of compounds through violent bubble collapse [23].

#### 3.2.2. Response Surface Optimization Experiments

The RSM results are shown in Table S7. The experimental data were fitted using a multiple regression model, resulting in the following quadratic polynomial regression equation:

(9) *Y* = 10.81 − 0.404*A* + 0.2624*B* − 0.1595*C* − 0.0754*AB* − 0.0259*AC* + 0.0424*BC* − 1.21*A*^2^ − 0.3139*B*^2^ − 0.8581*C*^2^

where *Y*, *A*, *B*, and *C* are the flavonoid yield, concentration of (NH_4_)_2_SO_4_, ultrasonic time, and ultrasonic temperature, respectively.

The quadratic regression model had a highly significant *p*-value (*p* < 0.01) (Table S8). Additionally, the adjusted coefficient of determination (*R*^2^_Adj_ = 0.995) suggested that there was minimal interference from uncontrolled factors, which indicates that this model can explain the majority of the variations in PFF production. The linear terms (*A*, *B*, and *C*) and quadratic terms (*A*^2^, *B*^2^, and *C*^2^) exhibited highly significant effects on the flavonoid yield (*p* < 0.01), and the interaction term *AB* showed a significant effect (*p* < 0.05). The extraction efficiency of flavonoid compounds initially increased with increases in the ultrasonication time and (NH_4_)_2_SO_4_ concentration but then decreased (Figure 2F and Figure S2B). The steep curvature of the *AB* interaction response surface indicated a strong synergistic effect between these two factors, which was consistent with the ANOVA results.

#### 3.2.3. Model Validation

According to the response surface methodology, the optimum conditions for ATPE were (NH_4_)_2_SO_4_ concentration of 17.8%, ultrasonication time of 12 min, and ultrasonication temperature of 44 °C. Verification experiments conducted in triplicate under these conditions resulted in flavonoid extraction yields of 10.787, 10.990, and 10.889 mg/g, with a mean value of 10.889 mg/g. This mean value was close to the theoretical value (relative error < 0.1%), which demonstrates there was excellent agreement between the experimental results and model predictions. The flavonoid extraction yields were higher than those reported by Zhu et al. for jujube peel [24]. Overall, the UA-ATPE efficiency was superior to that of conventional extraction methods.

### 3.3. Optimization of PFF Purification Using Macroporous Resins

#### 3.3.1. Screening of Macroporous Resins

**Figure 3:**
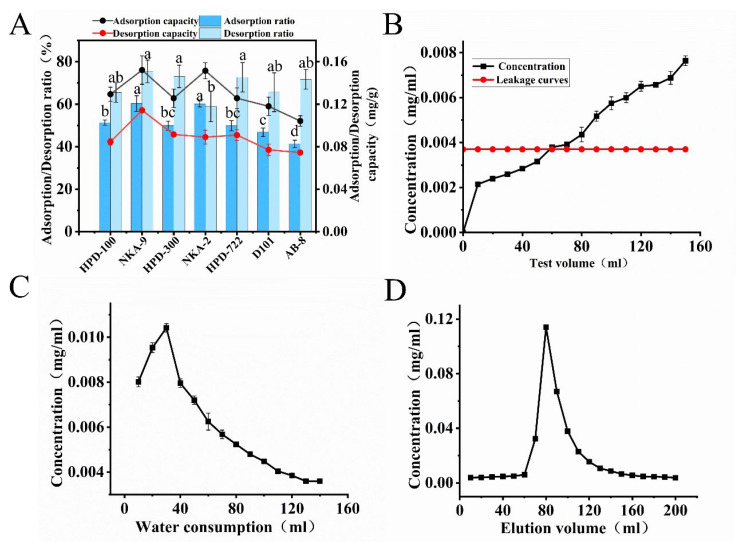
Screening of macroporous resins and optimization of single-factor conditions were performed. (**A**) The adsorption and desorption properties of different macroporous resins for PFF. (**B**) Formulation of the leakage curve. (**C**) Determination of the volume of the washing liquid. (**D**) Determination of the volume of the elution solution. The presence of different letters indicates that the observed differences are statistically significant (*p* < 0.05).

The physicochemical properties of all investigated macroporous resins were summarized in Table S9. Large differences in the adsorption/desorption performance were observed among the resins (Figure 3A). NKA-9 had the highest adsorption and desorption rates. This may be because NKA-9 is a polar resin with a large average diameter and a strong ability to adsorb flavonoids. Therefore, NKA-9 resin was selected as the macroporous resin for subsequent purification of total flavonoids from *P. tomentosa* flowers.

#### 3.3.2. Determination of the Leakage Point, Washing Volume, and Elution Profile

The flavonoid concentration in the effluent increased progressively with increases in the loading volume (Figure 3B). The leakage point, which was defined as 10% of the feed concentration [25], 0.037 mg/mL), was identified when the effluent concentration reached 0.0037 mg/mL at a loading volume of 60 mL. The relationship between washing volume and flavonoid concentration (Figure 3C) showed that the concentration equilibrium was achieved at a washing volume of 130 mL, which was selected as the optimum value. The elution profile (Figure 3D) showed a sharp flavonoid concentration peak at an eluent volume of 80 mL. Complete elution (effluent concentration approaching baseline) occurred at 150 mL, and this volume was the operational endpoint.

#### 3.3.3. One-Factor Experiments

**Figure 4:**
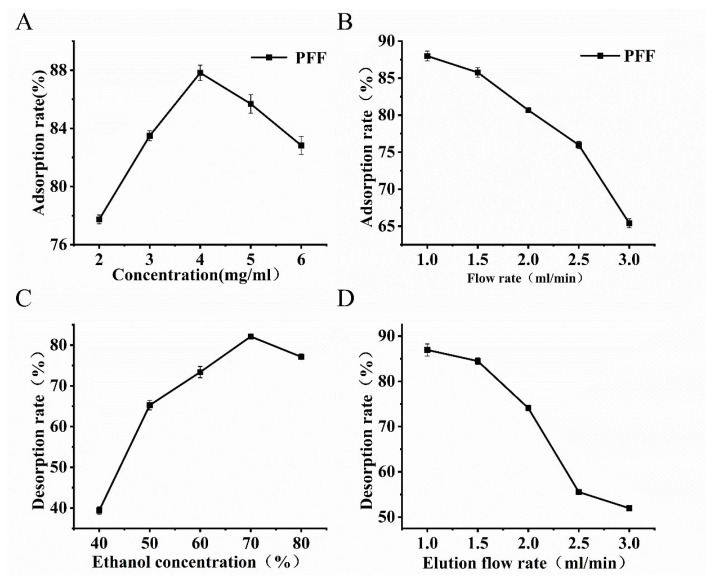
Optimization of adsorption/desorption conditions for the purification of PFF by NKA-9. (**A**) The influence of loading concentration on adsorption rate. (**B**) The influence of sample injection rate on adsorption rate. (**C**) The influence of ethanol concentration on the desorption rate. (**D**) The influence of elution rate on desorption rate.

The adsorption rate of NKA-9 resin exhibited concentration-dependent behavior, peaking at 4 mg/mL, and the flavonoids gradually filled all sites on the resin, which resulted in a gradual decrease in the adsorption rate [26]. Elevated flow rates reduced adsorption efficiency (Figure 4B); this change was potentially caused by insufficient contact time between the flavonoids and macroporous resin at higher flow rates [27]. Considering both the adsorption performance and time efficiency, a flow rate of 1.5 mL/min was selected as optimum. The desorption rate initially increased and then decreased with increases in the ethanol concentration (Figure 4C). Ethanol concentrations below 70% were insufficient for competition for resin adsorption sites and resulted in poor desorption [28]. Consequently, 70% ethanol was selected as the eluent. The maximum desorption efficiency (86.92%) was achieved at a flow rate of 1 mL/min (Figure 4D). Considering the desorption performance and time efficiency, a flow rate of 1.5 mL/min was selected as optimum.

#### 3.3.4. Purification Effect of Macroporous Resin

The purification effects of the macroporous resins were evaluated by comparing total flavonoid contents between crude extracts and purified fractions (Table S10). Under the optimized dynamic adsorption conditions, a purified product with a flavonoid content of 159.25 mg/g was given (as quantified by UV spectrophotometry). This value was 3.7 times that in the crude extract, which showed that the resin had a good purification effect. In a previous study by Yang et al., AB-8 resin was used to purify flavonoids from white tea, resulting in a 2.61-fold increase in flavonoid purity compared to the crude extract [9]. In the present study, NKA-9 resin was employed for the purification of flavonoids from *Paulownia tomentosa* flowers, achieving a 3.7-fold enhancement in purity, which was superior to the results reported by Yang et al.

### 3.4. UV–Vis and FT-IR Characterization

**Figure 5:**
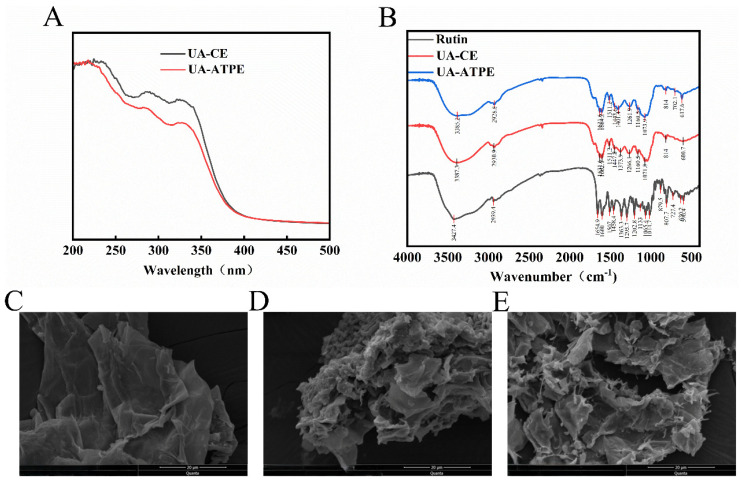
UV–vis (**A**) and FT-IR (**B**) spectra of extracts from different extraction methods and scanning electron micrograph of the effect of different treatments on the surface microstructure of *P. tomentosa* flowers. SEM images of (**C**) untreated control sample, (**D**) sample processed by UA-CE, and (**E**) sample processed by UA-ATPE.

The total flavonoids purified by UA-CE and UA-ATPE exhibited similar UV-Vis absorption profiles (Figure 5A). The chromatograms revealed two characteristic absorption peaks at 285 and 324 nm for both purification methods. Majority of flavones and flavonols have two major absorption bands that lie in the ranges of 300–400 and 240–285 nm [29]. These spectral characteristics were consistent with the typical absorption patterns of flavonoid compounds.

FTIR spectroscopy provided structural and functional group insights for understanding extraction mechanisms. The FTIR spectra of the rutin standard, UA-CE product, and UA-ATPE product were compared in Figure 5B. All purified products exhibited fundamental structural characteristics of flavonoids, and their spectral profiles were consistent with the rutin standard. In the functional group region, a peak for intermolecular hydrogen-bonded O-H stretching vibrations [30] was observed at 3427.4 cm^−1^ for the rutin standard, 3387.3 cm^−1^ for the UA-CE product, and 3385.2 cm^−1^ for the UA-ATPE product. The lower wavenumbers for the purified products suggest that stronger intermolecular hydrogen bonds form during extraction. Asymmetric -CH_2_- stretching vibrations [31] were detected at 2939.4 cm^−1^ (rutin standard), 2930.9 cm^−1^ (UA-CE product), and 2928.8 cm^−1^ (UA-ATPE product), with relatively weak absorption intensities indicating fewer hydrogens on saturated carbons. The spectrum of the UA-ATPE product was similar to that of the UA-CE product but had a low wavenumber for O-H stretching, which indicated that it had a more robust hydrogen-bonding network.

### 3.5. SEM Analysis

The microstructures of powders obtained from *P. tomentosa* flowers for the control (untreated), UA-CE, and UA-ATPE were directly observed using SEM. The untreated powder had an intact and smooth surface (Figure 5C). By contrast, the UA-CE powder showed pronounced wrinkling, surface roughening, and fissure formation (Figure 5D), which indicated the structure was compromised. Notably, the UA-ATPE powder showed severe surface erosion with large grooves (Figure 5E). The structural degradation originated from ultrasonic cavitation effects. Imploding cavitation bubbles generated localized mechanical forces on the surface of the *P. tomentosa* flower powder, and synergistic shear forces during extraction disrupted plant tissues. These forces enhanced flavonoid dissolution [32]. Crucially, comparative analysis revealed the UA-ATPE induced more pronounced structural disruption than UA-CE, which corroborated its superior efficacy in flavonoid release from the cells. These findings showed that both UA strategies effectively enhanced flavonoid extraction through targeted physical disintegration of *P. tomentosa* flower microstructures. A positive correlation between the extent of structural disruption in plant tissues and extraction efficiency is demonstrated by studies [33]. The improvement in extraction efficiency is driven by ultrasonication-induced particle size reduction and increased specific surface area, which in turn enhances the release of intracellular substances [34].

### 3.6. UPLC-MS/MS Analysis

**Figure 6:**
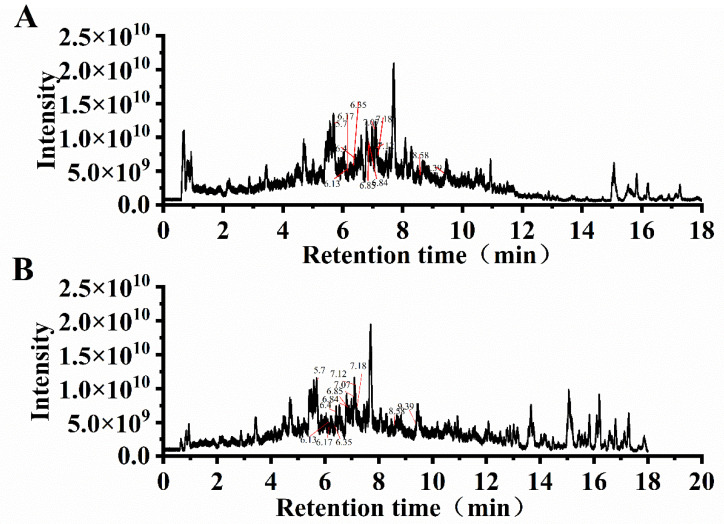
Total ion chromatograms in positive and negative ionization modes. (**A**) The total ion chromatogram of UA-CE. (**B**) The total ion chromatogram of UA-ATPE.

The flavonoid compositions of the purified *P. tomentosa* flower extracts were characterized using UPLC-MS/MS. Total ion chromatograms obtained in positive and negative ionization modes are presented in Figure 6 for UA-CE (Figure 6A) and UA-ATPE (Figure 6B) products. Sixteen major flavonoids were identified in the UA-CE product according to their retention times, mass-to-charge ratios, and other information (Table 1), comprising seven flavonoid compounds and nine flavonol compounds. A total of 17 major flavonoids were identified in the UA-ATPE product (Table 1), consisting of eleven flavonoid compounds and six flavonol compounds. Through compositional analysis of the purified extracts obtained by the two extraction methods, the predominant flavonoid compounds in UA-CE were identified as apigenin-7-glucuronide (777.984 ng/mg), kaempferol 3-O-sophoroside (363.579 ng/mg), and scutellarin (291.460 ng/mg), while in UA-ATPE, the major flavonoids were determined to be apigenin-7-glucuronide (767.816 ng/mg), apigenin (490.910 ng/mg), and scutellarin (276.242 ng/mg). The flavonoids identified in *Paulownia* flowers from previous studies were primarily apigenin and quercetin, along with luteolin, kaempferol, and glycosylated derivatives of apigenin [35,36,37]. This study first identified several flavonoid compounds in *P. tomentosa* flowers, including scutellarin, ombuin, robinetin and astragalin, which had not been previously reported in this plant species. Among these, scutellarin was found to be the most abundant flavonoid, with concentrations of 291.460 ng/mg in UA-CE and 276.242 ng/mg in UA-ATPE extracts.

### 3.7. Inhibition of α-Glucosidase by PFF and Flavonoid Monomer Substances

**Figure 7:**
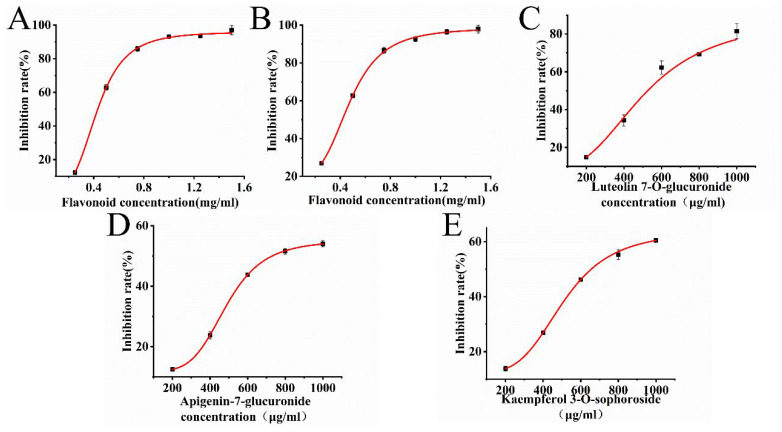
Evaluation of the inhibitory activity of PFF and flavonoid monomer substances against α-glucosidase. (**A**) Inhibitory activities against α-glucosidase of UA-CE-extracted PFF. (**B**) Inhibitory activities against α-glucosidase of UA-ATPE-extracted PFF. (**C**) Inhibitory activities against α-glucosidase of luteolin 7-O-glucuronide. (**D**) Inhibitory activities against α-glucosidase of apigenin-7-glucuronide. (**E**) Inhibitory activities against α-glucosidase of kaempferol 3-O-sophoroside.

The α-glucosidase inhibitory activity first increased with increases in the total flavonoid concentration and then gradually leveled off as the reaction approached equilibrium (Figure 7A,B). Significant α-glucosidase inhibitory activity was observed in PFF extracted by UA-CE and UA-ATPE, with IC_50_ values of 0.436 mg/mL and 0.412 mg/mL, respectively. At a concentration of 1 mg/mL, both extracts demonstrated inhibition rates exceeding 90%. However, their inhibitory efficacy was found to be slightly lower than that of acarbose. Notably, superior α-glucosidase inhibition was observed with UA-ATPE compared to UA-CE. Our results were consistent with previous research that showed flavonoids had great potential as inhibitors of α-glucosidase [38]. Compared with Scutellaria baicalensis, an inhibition rate of 81.76% against α-glucosidase was observed at 2 mg/mL. In contrast, an inhibition rate exceeding 90% was demonstrated by PFF at a lower concentration of 1 mg/mL, indicating its superior inhibitory potency [39]. Therefore, PFF was considered to have significant potential as a novel natural α-glucosidase inhibitor with relatively minor side effects. As shown in Figure 7C–E, the inhibition rates against α-glucosidase were gradually increased with elevated concentrations of the three flavonoids. However, apigenin-7-glucuronide and kaempferol 3-O-sophoroside were found to exhibit weaker inhibitory activity against α-glucosidase, with IC50 values of 0.809 mg/mL and 0.713 mg/mL, respectively. In contrast, luteolin 7-O-glucuronide demonstrated significant α-glucosidase inhibitory activity, with IC50 value of 0.508 mg/mL, indicating it to be one of the primary active constituents responsible for α-glucosidase inhibition in PFF.

### 3.8. Reversibility of Inhibition of α-Glucosidase by PFF and Luteolin 7-O-Glucuronide

**Figure 8:**
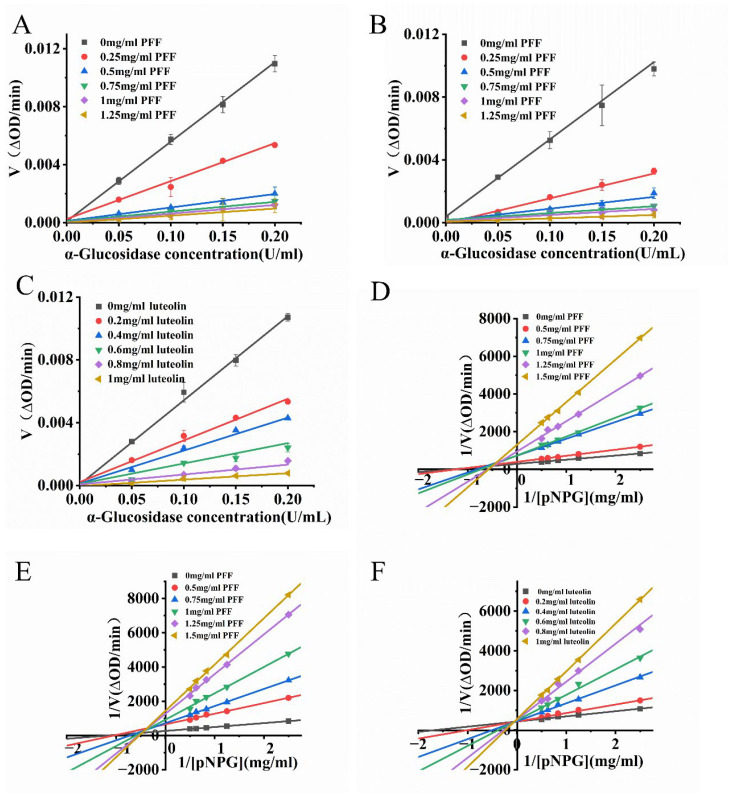
Investigation of the inhibition mechanism and type of PFF and luteolin 7-O-glucuronide against α-glucosidase. (**A**) The α-Glucosidase inhibition mechanisms of UA-CE-extracted PFF. (**B**) The α-Glucosidase inhibition mechanisms of UA-ATPE-extracted PFF. (**C**) The α-Glucosidase inhibition mechanisms of luteolin 7-O-glucuronide. (**D**) The α-Glucosidase inhibition type of UA-CE-extracted PFF. (**E**) The α-Glucosidase inhibition type of UA-ATPE-extracted PFF. (**F**) The α-Glucosidase inhibition type of luteolin 7-O-glucuronide.

The reversibility of α-glucosidase inhibition by PFF obtained through UA-CE (Figure 8A) and UA-ATPE (Figure 8B) was systematically investigated. There was a linear relationship between the initial reaction rate and the enzyme concentration, which passed through the origin. These results suggest that the inhibition of α-glucosidase by PFF is reversible. As shown in Figure 8C, the initial reaction rate was affected by luteolin 7-O-glucuronide concentration in a linear relationship passing through the origin, indicating that the inhibition of α-glucosidase by luteolin 7-O-glucuronide was reversible. This reversible inhibition suggested that luteolin 7-O-glucuronide was able to bind to α-glucosidase through weak non-covalent interactions (hydrophobic forces or hydrogen bonds), resulting in decreased enzymatic activity. These results were consistent with the findings reported by Hamed et al., where the flavonoids extracted from Moringa oleifera leaves were investigated and were found to exhibit potent inhibitory activity against α-glucosidase, with the inhibition further demonstrated to be reversible [40].

### 3.9. Type of Inhibition of α-Glucosidase by PFF and Luteolin 7-O-Glucuronide

Lineweaver–Burk plots for α-glucosidase inhibition by PFF extracted via UA-CE (Figure 8D) and UA-ATPS (Figure 8E) showed the slope of the line increased with increases in the PFF concentration. All lines intersected in the second quadrant. Combined with the kinetic parameters (Table S11), the reciprocal plots of 1/pNPG versus 1/*V* demonstrated strong linear correlations. Notably, the apparent *K*_m_ values increased with higher PFF concentrations, whereas the *V*_m_ values gradually decreased. These kinetic patterns align with characteristic features of mixed competitive inhibition; therefore, the PFF extracted by both UA-CE and UA-ATPS was a mixed competitive inhibitor of α-glucosidase. As shown in Figure 8F, all lines were observed to intersect in the second quadrant, with the Km value increasing and the Vm value decreasing with elevated concentrations of luteolin 7-O-glucuronide, indicating that luteolin 7-O-glucuronide acts as a mixed-competitive inhibitor of α-glucosidase. These results were consistent with those reported by Dlamini et al. for apigenin and pinocembrin isolated from *Scutellaria barbata* and by Mo et al. for four flavonoid glycosides containing coumaroyl or feruloyl groups obtained from *Ginkgo biloba* male flowers, where the inhibition of α-glucosidase was also demonstrated to be of mixed-competitive type [41,42].

## 4. Conclusions

This study successfully achieved its primary objective of developing an efficient and sustainable method for the extraction of bioactive flavonoids from *Paulownia tomentosa* flowers (PFF) with high α-glucosidase inhibitory activity. A systematic comparison between ultrasound-assisted cellulase extraction (UA-CE) and ultrasound-assisted aqueous two-phase extraction (UA-ATPE) confirmed the superior efficacy of the latter. Under the optimized conditions of 17.8% (NH_4_)_2_SO_4_, 12 min ultrasonication at 44 °C, UA-ATPE achieved a high PFF yield of 10.889 mg/g.

The ultrasound-assisted aqueous two-phase extraction process developed in this study demonstrates potential for industrial-scale application, with its core equipment being standardized in both traditional Chinese medicine and chemical industries. Compared to organic solvent methods, this process offers combined economic and environmental benefits through recyclable reagents, lower energy consumption, reduced water usage, and minimal emissions, aligning with green pharmaceutical manufacturing requirements. The α-glucosidase inhibitory activity of Paulownia tomentosa flower flavonoids is closely related to their structural characteristics. The activity of luteolin 7-O-glucuronide confirms that the ortho-dihydroxy groups on the B-ring serve as crucial pharmacophores, while the reduced activity of ombuin results from steric hindrance introduced by the glycosyl group at the 7-position. However, this study has certain limitations. The biological validation was primarily conducted in vitro; future work should include in vivo assays to confirm the hypoglycemic efficacy and safety. Furthermore, the industrial feasibility analysis remains at a theoretical stage and requires validation through pilot-scale experiments.

In conclusion, this work not only provides an optimized, green extraction process for PFF but also offers fundamental insights into its inhibitory mechanism against α-glucosidase. The findings lay a solid foundation for the further development of *P. tomentosa* flower extracts as functional food ingredients or natural-based pharmaceuticals for managing postprandial hyperglycemia.:

## Figures and Tables

**Table 1:** Quantitative analysis of major flavonoid compounds in PFF extracted by UA-CE and UA-ATPE methods.

No.	Name	Class	UA-CE Content (ng/mg)	UA-APTE Content (ng/mg)	*m*/*z*	Retention Time (min)
1	Isorhamnetin-3-O-glucoside	Flavonols	31.26	19.15	479.12	7.18
2	Robinetin	Flavonols	17.91	9.32	303.05	6.17
3	Rutin hydrate	Flavonols	40.42	16.01	611.16	6.35
4	Rutin	Flavonols	40.42	16.01	611.16	6.35
5	Astragalin	Flavonols	55.64	41.94	449.11	7.12
6	Quercitrin	Flavonols	56.03	42.23	449.11	7.07
7	Isoquercetin	Flavonols	71.06	34.81	465.10	6.4
8	Hyperoside	Flavonols	71.15	34.85	465.10	6.4
9	Cynaroside	Flavones	87.73	58.69	449.11	6.13
10	Apigenin 7-glucoside	Flavones	122.11	126.23	433.11	6.84
11	Luteolin	Flavones	255.45	219.70	287.06	8.58
12	Apigenin	Flavones	266.23	490.91	271.06	9.39
13	Luteolin 7-O-glucuronide	Flavones	284.44	269.59	463.09	6.13
14	Scutellarin	Flavones	291.46	276.24	463.09	6.17
15	Kaempferol 3-O-sophoroside	Flavonols	363.58	53.586	611.16	5.7
16	Apigenin-7-glucuronide	Flavones	777.98	767.82	447.09	6.85
17	Ombuin	Flavones	15.73	34.87	331.08	11.06
18	Diosmetin	Flavones	13.29	31.12	301.07	9.51
19	3′,4′,7-Trimethoxyquercetin	Flavonols	2.43	31.07	345.10	12.04
20	Chrysoeriol	Flavones	13.24	31.00	301.07	9.47
21	Hispidulin	Flavones	13.23	30.97	301.07	9.45

## Data Availability

The original contributions presented in this study are included in the article/Supplementary Materials. Further inquiries can be directed to the corresponding authors.

## Supplementary Materials

The following supporting information can be downloaded at https://www.mdpi.com/article/10.3390/foods14223941/s1, Figure S1: Rutin standard curve; Figure S2: Two-dimensional response surface plot. (A) The 2D response surface plot of UA-CE. (B) The 2D response surface plot of UA-ATPE; Table S1: Factors and levels for the single-factor experiments in UA-CE optimization; Table S2: Responsive surface design scheme of UA-CE; Table S3: Factors and levels for the single-factor experiments in UA-ATPE optimization; Table S4: Responsive surface design scheme of UA-ATPE; Table S5: Response surface results of UA-CE; Table S6: ANOVA for the fitted quadratic model of extraction of *P. tomentosa* flower flavonoids. Note: * indicates a significant difference at *p* < 0.05; ** indicates a highly significant difference at *p* < 0.01; Table S7: Response surface results of UA-ATPE; Table S8: ANOVA for the fitted quadratic model of extraction of *P. tomentosa* flower flavonoids; Table S9: Physical properties of all macroporous resins used in this work; Table S10: Purification effect of macroporous resins; Table S11: Kinetic equation of inhibition of α-glucosidase by PFF.
