# Supplementary material for: Bioactive Flavonoids from Paulownia tomentosa Flowers: Extraction Optimization and α-Glucosidase Inhibitory Kinetics
Source: Foods. 2025 Nov 18;14(22):3941. doi: 10.3390/foods14223941 (PMC12652004; doi:10.3390/foods14223941)
Supplement: Supplementary file 1 [file foods-14-03941-s001.zip › foods-3947411-supplementary.pdf]

Supplementary materials

Bioactive Flavonoids from *Paulownia tomentosa* Flowers: Extraction Optimization and α-Glucosidase Inhibitory Kinetics

Fu Jiang [1,†], Haibo Yang [1,†], Xiaoqiao Zhai [2,*], Zhenli Zhao [1] and Guoqiang Fan [1,*]

[1] Institute of Paulownia, Henan Agricultural University, Zhengzhou 450002, China
[2] Henan Province Academy of Forestry, Zhengzhou 450008, China
* Correspondence: user7117@163.com (X.Z.); zlxx64@126.com (G.F.)
† These authors contributed equally to this work.

Supplementary Figure legends

Supplementary Figure S1. Rutin standard curve.

Supplementary Figure S2. Two-dimensional response surface plot.

(A) The 2D response surface plot of UA-CE. (B) The 2D response surface plot of UA-ATPE.

Supplementary Figure S1

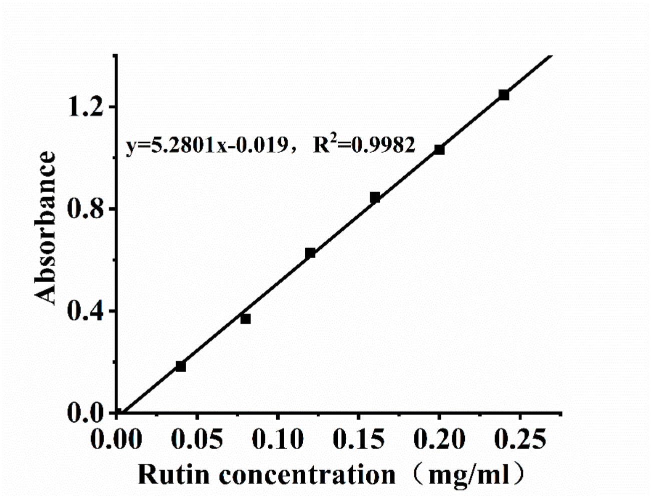

Supplementary Figure S2

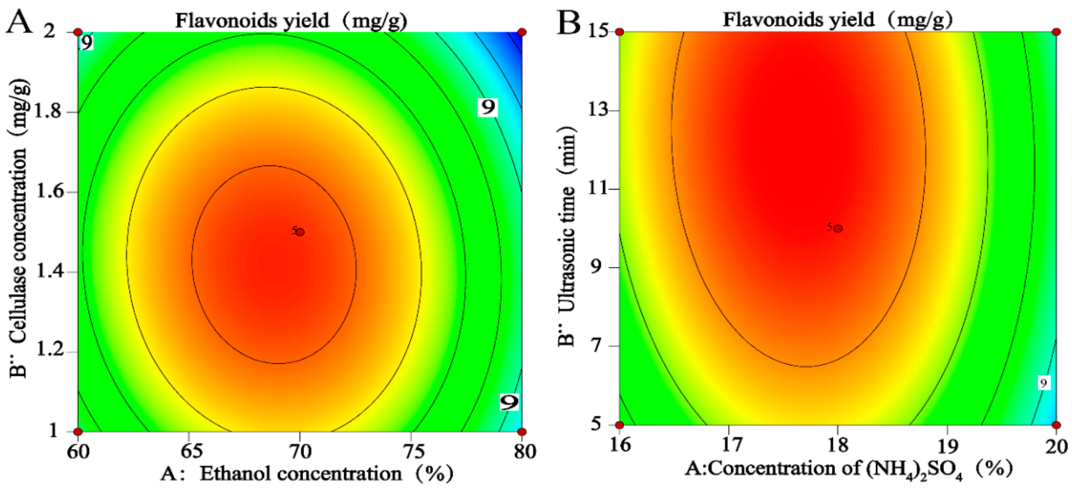

Supplementary Table legends

Supplementary Table S1. Factors and levels for the single-factor experiments in UA-CE optimization.

Supplementary Table S2. Responsive surface design scheme of UA-CE.

Supplementary Table S3. Factors and levels for the single-factor experiments in UA-ATPE optimization.

Supplementary Table S4. Responsive surface design scheme of UA-ATPE.

Supplementary Table S5. Response surface results of UA-CE.

Supplementary Table S6. ANOVA for the fitted quadratic model of extraction of P. tomentosa flower flavonoids.

Note: * indicates a significant difference at $P<0.05$; ** indicates a highly significant difference at $P<0.01$.

Supplementary Table S7. Response surface results of UA-ATPE.

Supplementary Table S8. ANOVA for the fitted quadratic model of extraction of P. tomentosa flower flavonoids.

Supplementary Table S9. Physical properties of all macroporous resins used in this work

Supplementary Table S10. Purification effect of macroporous resins.

Supplementary Table S11. Kinetic equation of inhibition of α-glucosidase by PFF.

Supplementary Table S1

Factor	Levels
Ethanol Concentration (%)	40, 50, 60, 70, 80
Cellulase Concentration (mg/g)	0.5, 1.0, 1.5, 2.0, 2.5
Digestion Time (h)	1.0, 1.5, 2.0, 2.5, 3.0
Digestion Temperature (°C)	30, 40, 50, 60, 70
Ultrasonication Time (min)	5, 10, 15, 20, 25

Supplementary Table S2

Level	A	B	C
-1	60	1	10
0	70	1.5	15
1	80	2	20

Supplementary Table S3

Factor	Levels
$(NH_4)_2SO_4$ Concentration (%)	16, 18, 20, 22, 24
Ultrasonication Time (min)	5, 10, 15, 20, 25
Ultrasonication Power (W)	160, 200, 240, 280, 320
Ultrasonication Temperature (°C)	35, 40, 45, 50, 55

Supplementary Table S4

Level	A	B	C
-1	16	5	40
0	18	10	45
1	20	15	50

Supplementary Table S5

No.	A	B	C	Y
1	80	2	15	8.52
2	80	1	15	8.84
3	60	1.5	20	9.25
4	70	1.5	15	9.94
5	70	1.5	15	9.88
6	70	2	10	9.14
7	70	1	10	9.39
8	70	2	20	9.25
9	70	1.5	15	9.86
10	60	1	15	9.06
11	80	1.5	10	8.78
12	70	1	20	9.58
13	60	1.5	10	9.17
14	80	1.5	20	9.00
15	70	1.5	15	9.82
16	70	1.5	15	9.85
17	60	2	15	8.95

Supplementary Table S6

Source	Sum of squares	DF	Mean squares	F	P	
Model	3.12	9	0.3471	204.51	<0.0001	Significant
A	0.2094	1	0.2094	123.34	<0.0001	**
B	0.1275	1	0.1275	75.14	<0.0001	**
C	0.0450	1	0.0450	26.49	0.0013	**
AB	0.0105	1	0.0105	6.20	0.0416	*
AC	0.0050	1	0.0050	2.97	0.1284	
BC	0.0016	1	0.0016	0.9172	0.3701	
A^2	1.83	1	1.83	1080.91	<0.0001	**
B^2	0.5706	1	0.5706	336.17	<0.0001	**
C^2	0.1118	1	0.1118	65.87	<0.0001	**
Residual	0.0119	7	0.0017			
Lack of Fit	0.0031	3	0.0010	0.4735	0.7174	Not significant
Pure Error	0.0088	4	0.0022			
Cor Total	3.14	16				
			R^2=0.9962		R^2_{Adj}=0.9913	

Supplementary Table S7

No.	A	B	C	Y
1	18	10	45	10.89
2	18	5	50	9.16
3	18	10	45	10.89
4	16	5	45	9.38
5	18	15	50	9.80
6	18	15	40	10.04
7	16	10	50	9.01
8	20	10	40	8.54
9	20	15	45	9.05
10	18	10	45	10.72
11	18	10	45	10.82
12	18	5	40	9.57
13	16	10	40	9.27
14	20	10	50	8.17
15	18	10	45	10.75
16	16	15	45	10.03
17	20	5	45	8.71

Supplementary Table S8

Source	Sum of squares	DF	Mean squares	F	P	
Model	12.62	9	1.40	359.69	<0.0001	Significant
A	1.31	1	1.31	334.97	<0.0001	**
B	0.5509	1	0.5509	141.34	<0.0001	**
C	0.2035	1	0.2035	52.20	0.0002	**
AB	0.0227	1	0.0227	5.83	0.0464	*
AC	0.0027	1	0.0027	0.6890	0.4339	
BC	0.0072	1	0.0072	1.84	0.2165	
A^2	6.16	1	6.16	1580.30	<0.0001	**
B^2	0.4149	1	0.4149	106.45	<0.0001	**
C^2	3.10	1	3.10	795.33	<0.0001	**
Residual	0.0273	7	0.0039			
Lack of Fit	0.0034	3	0.0011	0.1867	0.9003	Not significant
Pure Error	0.0239	4	0.0060			
Cor Total	12.65	16				
			R^2=0.9978		R^2_{Adj}=0.9951	

Supplementary Table S9

Model	Polarity	Specific surface area (m^2/g)	Average pore size (nm)
HPD100	Non-polar	650-700	8.5-9.0
NKA-9	Polar	500-550	10.0-12.0
HPD300	Non-polar	800-870	5.0-5.5
NKA-2	Polar	160-200	14.5-15.5
HPD-722	Non-polar	650-700	8.0-9.0
D101	Non-polar	500-550	9.0-10.0
AB-8	Weak polar	480-520	13.0-14.0

Supplementary Table S10

No.	Sample	Flavonoid yield（mg/g）	Mean（mg/g）	RSD（%）
1		43.56		
2	Flavonoid primitive	42.14	42.93	1.69
3		43.09		
4		157.67		
5	NKA-9 purified	160.51	159.25	0.91
6		159.56		

Supplementary Table S11

Extraction method	Concentration（mg/mL）	Regression equations	R^2	K_m	V_m
UA cellulase extraction	0	y=220.024x+292.486	0.9931	1.183	0.00083
	0.5	y=322.870x+398.001	0.9977	1.294	0.00056
	0.75	y=894.028x+726.476	0.9995	2.055	0.00019
	1	y=1003.288x+735.307	0.9968	2.370	0.00016
	1.25	y=1603.802x+947.195	0.9947	2.927	0.00009
	1.5	y=2258.417x+1303.941	0.9990	3.079	0.00007
UA-ATPE	0	y=228.917x+264.930	0.9984	1.395	0.00079
	0.5	y=621.626x+650.031	0.9948	1.540	0.00029
	0.75	y=1000.210x+717.868	0.9992	2.390	0.00016
	1	y=1539.393x+921.468	0.9939	2.852	0.00010
	1.25	y=2319.939x+1264.387	0.9984	3.181	0.00006
	1.5	y=2707.282x+1433.905	0.9987	3.324	0.00005
